# Gender shift in realisation of preferred type of gp practice: longitudinal survey over the last 25 years

**DOI:** 10.1186/1472-6963-7-111

**Published:** 2007-07-13

**Authors:** Tanja Maiorova, Fred Stevens, Lud van der Velden, Albert Scherpbier, Jouke van der Zee

**Affiliations:** 1 Institute of Medical Education, Faculty of Medicine, University of Maastricht, The Netherlands; 2 Department of Health Care Studies, University of Maastricht, The Netherlands; 3 NIVEL, Netherlands Institute of Health Services Research, Utrecht, The Netherlands; 4 Institute of Medical Education, Faculty of Medicine, University of Maastricht, The Netherlands; 5 NIVEL, Netherlands Institute of Health Services Research, Utrecht and Department of Health Care Studies, University of Maastricht, The Netherlands

## Abstract

**Background:**

An increasing number of newly trained Dutch GPs prefer to work in a group practice and as a non-principal rather than in a single-handed practice. In view of the greater number of female doctors, changing practice preferences, and discussions on future workforce problems, the question is whether male and female GPs were able to realise their initial preferences in the past and will be able to do so in the future.

**Methods:**

We have conducted longitudinal cohort study of all GPs in the Netherlands seeking a practice between 1980 and 2004. The Netherlands Institute of Health Services Research (NIVEL) in Utrecht collected the data used in this study by means of a postal questionnaire. The overall mean response rate was 94%.

**Results:**

Over the past 20 years, an increasing proportion of GPs, both male and female, were able to achieve their preference for working in a group practice and/or in a non-principal position. Relatively more women than men have settled in group practices, and more men than women in single-handed practices; however, the practice preference of men and women is beginning to converge. Dropout was highest among the GPs without any specific practice preference.

**Conclusion:**

The overwhelming preference of male and female GPs for working in group practices is apparently being met by the number of positions (principal or non-principal) available in group practices. The preference of male and female GPs regarding the type of practice and job conditions is expected to converge further in the near future.

## Background

Several decades ago, the single-handed practice was the common European GP setting. Through the years, this has changed in favour of group practices [1]. In this study we analysed trends in the preference for group or single-handed practices, and the realisation of this preference, among newly qualified GPs in the Netherlands over the last 25 years.

Previous research indicated two important developments concerning the practice preferences of young GPs. First, younger generations of GPs prefer other working conditions and other practice settings than older GPs, settings that provide more opportunity for leisure time, flexibility, and the sharing of practice responsibilities [2]. Secondly, all over Europe a steady feminisation of general practice, and of the medical profession in general, is taking place [3-8]. The likely result is a reduction of GPs' working hours together with a stronger preference for working in a group practice, locum (non-principal) work, and opportunities to combine work and family life [8,9]. Although men used to be more motivated by financial incentives than women, newly trained male GPs also prefer to work less than full-time and increasingly support flexible office hours in order to accommodate family and home responsibilities [2,10]. Even though men and women may differ in their preferred form of medical practice, there are trends in common, namely, increasingly more recently qualified GPs prefer: (a) a group practice to a single-handed practice, (b) part-time over full-time employment [10-13] and (c) more preference for non-principal position.

In the light of these changing practice preferences, we do not know whether newly qualified GPs are and were able to realise their plans and initial preferences. There may be an increasing mismatch between the type of practice left by retiring GPs and the type preferred by newly trained ones. This mismatch could result in workforce problems if young GPs have to settle in a non-preferred type of practice, which may lead to the decision not to settle at all and to drop out [2,14-16].

Previous research into practice preference and realisation among newly qualified GPs was often confined to samples and/or to the analysis of one or a just a few yearly cohorts. In this study, we analysed trends in practice choices and realisation of preferences from 1980 onwards. Longitudinal data shed light on how the relation between preference and realisation has developed through the years. Insights into these long-term trends regarding the GP workforce in the Netherlands can contribute to our understanding of preferred careers of male and female GPs, and can help to better forecast the balance of need and supply of GPs in the future for other countries too.

We first analyse long-term trends in preferences for single and group practice and for principal and non-principal position. Then we investigate whether newly graduated GPs were able to realise their preference, and finally, what were the characteristics of those who dropped out of the profession. We addressed the following questions:

(a) How has the preference for single-handed and group practice and for principal and non-principal position developed over time among male and female GPs?

(b) Were GPs able to realise their practice preference and if so, was this similar for men and women?

(c) What preferences did the GPs have who have dropped out of the profession?

### GPs in the Netherlands

In the Netherlands, virtually the entire population is registered with a GP who provides primary care services and who is the gatekeeper to other, more specialised medical services. GPs in the Netherlands are largely self-employed, and nowadays most work in group practices. The percentage of GPs working in a group practice increased from 28% in 1980 to 73% (66% of all male GPs, 93% of all female GPs) in 2005 [17,18]. There has been a reduction in total numbers of GPs seeking a practice caused by the decreasing number of students entering general practice. The Netherlands has a rather low GP density, with the average GP list being 2053 patients in 1999, compared with 1663 patients in the UK, and 1523 patients as the European average [17,19,20].

Similar to GPs in the UK, Dutch GPs work 51 hours a week on average on a full-time basis. Dutch GPs who work as locum, or non-principal, in a group practice are usually employed by the GP who runs the practice. While GPs in the UK are usually reimbursed on a combination of allowance, capitation, and quality payments, Dutch GPs were (up till 2006) paid on capitation (sick fund patients) and on fee-for-service basis (privately insured patients); this private/public remuneration system was replaced by a mixed capitation fee-for-services system in 2006. No major change to remuneration of GPs in The Netherlands has taken place between 1980 and 2005.

## Methods

### Data

The data used in this study were collected by the Netherlands Institute of Health Services Research (NIVEL) in Utrecht, which collects data on all GPs in the Netherlands. In 1980 a first questionnaire was sent to all registered Dutch GPs to assess their actual employment status. In subsequent years, questionnaires were sent to only those who had recently qualified. They were asked to fill in the questionnaire in January, the first year following qualification. GPs kept receiving a similar questionnaire every year, until they finally had settled in a general practice, or had given up finding a suitable position (abstained). Topics in the questionnaire were the person's sex, practice type preference, and current position (working in a group or in a single-handed practice, a principal or a non-principal position). Through the years, the overall mean response rate was fairly stable about 94%.

The number of practice-seeking GPs each year includes both who have qualified the same year and have been looking for a position for more than one year.

### Variables

The preference for practice type was defined as the number of GPs who were looking for a position in (a) a single-handed practice, (b) a group practice, (c) a non-principal position or (d) had no particular practice type preference.

Realisation of preference was defined as the percentage of practice-seeking GPs who had found a position consistent with their initial practice choice. The possible types of settlement were: (a) a principal in a single-handed practice, (b) a principal in a group practice, (c) a non-principal and (d) did not settle at all.

### Analysis

A descriptive, longitudinal analysis was conducted, including almost the complete population of GPs graduated between 1980 and 2004.

As the data set was not a sample, but included the whole population of GPs who qualified between 1980 and 2004, we abstained from statistical testing. We analysed long-term realisation of practice preference merely among GPs who qualified in 1980 to 1999. It can take several years before GPs finally find a permanent position in a practice. On this reason we considered that the data for GPs who qualified in 2000 to 2004 would not accurately reflect the realisation of practice preferences.

## Results

### Preferences of practice-seeking GPs

**Figure 1:**
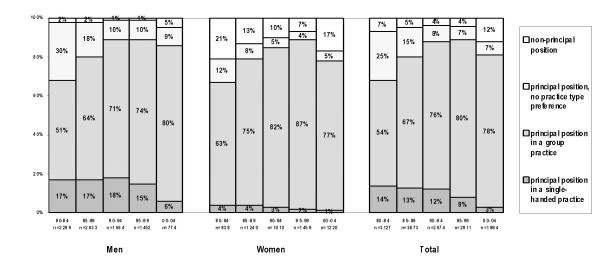
Practice type preference by Dutch GPs

For many years, the preference for a single-handed practice among male GPs was stable at about 17%, and only in the last 5 years did this preference drop to 6% (see Figure 1). Preference for a principal position in a group practice increased from 51% early 1980s to 80% more recently. A substantial proportion of male GPs had no particular preference, or was undecided about this in the 1980s (30%). This has since changed, with only 10% of male GPs expressing no preference for working in a single/group practice from 1995 onwards. A non-principal position preference was negligent and increased up to 5% in 2000–04.

Similar trends were observed among female GPs for single-handed practices and for non-principal positions. Single-handed practice was never popular among female GPs, and the proportion of women seeking such practices declined from 4% to 1% over the study period. There was and is a strong preference for working as a principal in a group practice: 63% of female GPs preferred this type of practice in the early 1980s and 87% in the period 1995–99. The proportion of female GPs without a specific preference dropped from 12% to 5%, most likely in favour of a preference for working in a group practice. The proportion of women favouring a non-principal position diminished at first, and has increased in 2000–04 to 17%.

### Realisation of practice preference: GPs with a preference for a principal position

**Figure 2:**
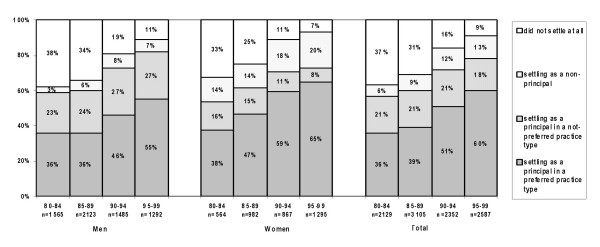
Realised practice type, for Dutch GPs preferring a position as a principal.

Figure 2 shows the total number of GPs who were looking for a principal position in either a single-handed or in a group practice. In the 80s 36 % of GPs who were looking for a principal position in a single-handed or a group practice was able to realise their preference. This percentage has increased to 60% in the period 1995–99. From 1985 on, women were slightly more successful than men in realising their preferences. There is a consistently increasing percentage of GPs, higher among women than among men (20% versus 7% in 1995–99), who settled as a non-principal, while looking for a principal position. The share of GPs who did not settle at all had fallen from 37% in 1980–84 to 9% in 1995–99. Relatively more male than female dropped out of the profession.

### Realisation of practice preference: GPs with a preference for a principal position in a single-handed practice

**Figure 3:**
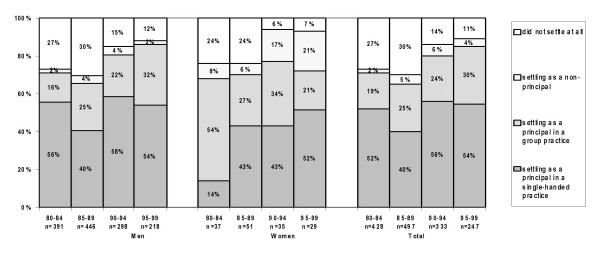
Realised practice type, for Dutch GPs preferring a position as a principle in a single-handed practice.

Figure 3 shows the proportion of GPs who initially preferred to work in a single-handed practice. The mean proportion of male GPs who realised their preference fluctuated between 1980 and 1999, with an average of 55% (min 34%, max 72%). The percentage of female GPs who realised their preference for a single-handed practice increased from 14% (1980–84) to 52% (1995–99). Women tended to work in a group practice. Their preference may have changed in favour of a group practice or they may still prefer a single-handed practice but have opted for a second-best solution. This trend was also observed among men: in 1980–1984 16% had a position in a group practice and in 1995–99 32%. Relatively more women than men had a locum (non-principal) position in a practice, and this proportion increased from 8% (1980–84) to 21% (1995–99).

The percentage of the GPs who dropped out of the profession was initially rather high and decreased for both men (from 27% to 12%) and women (from 24% to 7%) in the last decade.

### Realisation of practice preference: GPs with a preference for a principal position in a group practice

**Figure 4:**
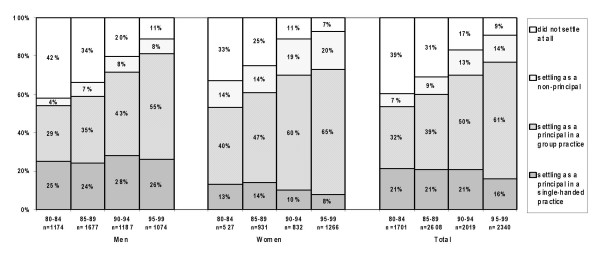
Realised practice type, for Dutch GPs preferring a position as a principal in a group practice.

Among GPs who expressed a preference for working in a group practice as a principal, female GPs were more likely than male GPs to find a position in such a practice; however, over the years the proportions have become more equal: 55% male and 65% female (figure 4). The proportion of men who settled in a group practice has continued to increase from 29% in 1980–84 to 55% in 1995–99. Nowadays, the proportion of female GPs who have a non-principal position has increased, from 14% in 1980–84 to 20% in 1995–99.

The number of dropouts among those who preferred a group practice was at first rather high (42% for men and 33% for women). This percentage has declined rapidly in the period between 1995 and 1999 (11% and 7% respectively).

### Realisation of practice preference: GPs with a preference for a principal position without practice type preference

**Figure 5:**
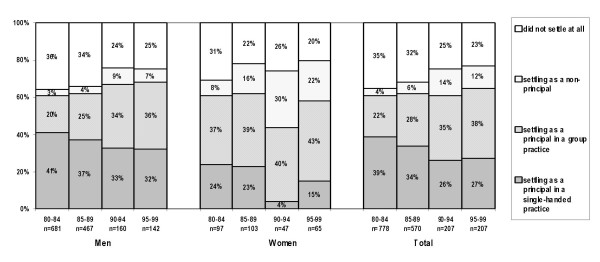
Realised practice type, for Dutch GPs preferring a position as a principal without preference for the practice type.

The trends are similar to GPs who had particular preferences. A higher proportion of both men and women have settled in a group practice in recent years (figure 5). Relatively more men than women worked in a single-handed practice, and relatively more women than men settled in a group practice and held a non-principal position.

The dropout rate was higher among both male and female GPs who had no expressed preference for working in a single-handed or group practice than it was among GPs who had such a preference. Although the dropout rate has decreased over the past 20 years, it is still relatively high: 25% for men and 20% for women in 1995–99.

### Realisation of practice preferences: GPs seeking a non-principal position

**Figure 6:**
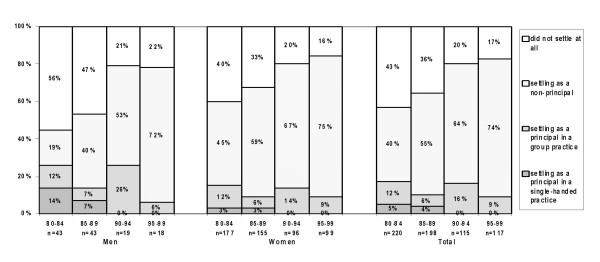
Realised practice type, for Dutch GPs seeking a position as a non-principal.

Increasing percentage of GPs (higher among women than men) settled as a non-principal according to their preference: 40% in the early 1980s and 75% in the late 1990s (figure 6). Through the years, there was a higher percentage GPs who settled as a principal in a group practice than in a single-handed practice. The share of GPs who did not settle has significantly decreased.

## Discussion

In this study we investigated long-term trends in the newly qualified GPs concerning the following issues: (a) GP preference for working in a single-handed or group practice, principal or non-principal position (b) realisation of this preference, (c) the destination of GPs without a specific preference for practice type, and (d) drop-out.

Our findings show that over the last 25 years the preference for working in a single-handed practice has strongly decreased so as to be virtually extinct, not only among female GPs but also among male GPs. The preference among male GPs for working in a group practice either as a principal or non-principal has increased steadily over the years, such that now both male and female GPs have an overwhelming preference for working in a group practice. There could be a number of explanations for this perspective. Firstly, currently, Dutch GPs choose often a sub-specialisation to offer an enhanced care and take over some tasks from the hospital physicians. This aim is easier to reach when joining together in a group practice. Secondly, present need for more time for family life applies not only to women but also to men. A study from Canada showed that female and male physicians do not differ significantly in their work hours or orientation towards patient care [21]. Although in the Netherlands, men still work longer hours than women, this development could take place in the future. Another explanation may involve gender identity, which has become an issue in recent years. The stereotypes of typical male and female physicians' orientations towards job arrangements have changed. Even though men and women still hold different work and family values and interests, the gap between their lifestyle attitudes has become smaller. Consequently, for the coming years we expect that (a) group practices will continue to be the most favoured practice setting among GPs seeking to establish themselves in a practice, and (b) male and female preference for this type of practice will continue to converge.

More women than men hold a non-principal position, although the proportion of both male and female GPs who have such positions increased over the study period. Similar results were obtained in a Scottish study [8]. With a few exceptions, men realise positions more frequent in a single-handed practice, and women in a group practice, regardless of their original practice choice. This was also true for GPs who had no specific preference for a practice type. It seems that women choose jobs that best facilitate achieving a satisfactory work-family balance and/or that they are more flexible than men regarding the choice of practice. Our finding that female GPs are also more likely to hold a non-principal position in a group practice is in line with this. Further research into gender differences in practice setting is needed.

We found that the number of GPs who dropped out has decreased steadily over the years. Drop-out rates remained more stable in those GPs without a specific preference. The lack of a clear preference for a specific type of practice continues to form a serious risk of dropping out. In the 1980s dropout rates (that is not settling as a GP at all) were rather high (between 30% and 40%) and did not differ much between categories of GPs (males, females, with and without preference for a specific type of practice). This changed in the 1990s, with there being a decrease in dropout rates among GPs with an expressed preference for a type of practice. Dropout rates remained stable in those GPs without a specific preference. This finding somewhat contradicts expectations, because one would suppose that GPs without a preference would be more flexible in taking a position in a certain type of practice. However, as became evident from our results, this group is, surprisingly, more inclined to step out of the profession. The most frequent job alternatives were an occupational physician and a nursing home physician [13].

A question is whether these Dutch developments are relevant to other European countries. We believe that this to be the case: the proportion of female doctors is increasing all over Europe [21]. The overall influx of women into medicine has highlighted issues concerning flexibility and the work-family balance. It is also evident that a single-handed practice is unsuitable for combining work and family duties. This might become a problem in countries with a high proportion of single-handed practices, and may lead to decreased general practice coverage and to a shortage in GPs.

Will a mismatch in demand and supply of practices result in a workforce crisis? Recent evidence shows that in England the number of GPs increased by 1.5% a year between 1994 and 2004, and at the same time there was a decrease in single-handed practices and an increase in larger group practices [6]. This shift is also taking place in the Netherlands. However, notwithstanding an increase in the availability of group practices in the Netherlands, similar to the UK situation, GPs are not distributed evenly across the country [22]. The number of male GPs seeking a position in a group practice is increasing rapidly, and more men are taking positions in group practices, even if they initially preferred a single-handed practice. Apparently, the number of vacant partnerships is also likely to increase. As the older generation of GPs reaches retirement, a number of practices will become vacant, so we do not envisage there being insufficient positions available. Given the demand for GP coverage, former single-handed practices may well be developed into group practices, to meet the current demand of newly qualified GPs for such positions, as found in this study. In the light of our research findings, a workforce crisis seems unlikely. Changing male and female GPs preferences for working in a group practice will go together with a greater availability of such practice settings. Any discrepancy between demand and supply will be mainly due to the uneven spread of GPs across the country. A far more serious problem, however, is the rather large number of trained GPs who do not find a position. Even though the number of dropouts has decreased over the years, this problem needs urgent attention and further investigation of its background.

We had access to a unique longitudinal data set, consisting of information about all GPs who qualified in the Netherlands over the last 25 years. The main limitation of our study is that recently qualified GPs who immediately found a position in a practice were not included, as they did not have to answer the questions on their preferences. This group counts on average 6%. Despite this limitation, our results substantiate previous concern about future qualitative and quantitative problems in the supply of GPs in relation to changing career preferences of male and of female doctors. It is important to take these changing work preferences of GPs into account in health care policies.

## Conclusion

Mismatches between GP practice preference and realisation have become less common in the last few years. Both male and female GPs have an overwhelming preference for working in a group practice and this preference is being met. The preference of male and female GPs regarding the type of practice and job conditions is expected to converge further in the future.

The friction between preference and realisation was more pressing in the 1980s, as a substantial dropout rate among newly qualified GPs might indicate. A considerable proportion of GPs are now able to realise their practice preference, while the dropout rate has decreased.

Though our data only show relations and do not allow for casual explanations, it seems that the 'yield' of the postgraduate GP training has improved. In the future, dropout rates could be reduced if Dutch medical schools were to pay attention to future GPs without a specific practice preference, as this may be an indicator of a lukewarm interest in the profession.

## Competing interests

The author(s) declare that they have no competing interests.

## Authors' contributions

TM, FS, AS, LvdV and JvdZ contributed equally to this paper. LvdV carried out the analysis. TM, FS, AS, LvdV and JvdZ pictured the idea and drafted the manuscript. All authors have read and approved the final manuscript.

## Pre-publication history

The pre-publication history for this paper can be accessed here:


